# Association of body mass index with surgical complications after minimally invasive hysterectomy

**DOI:** 10.1007/s00404-025-08073-9

**Published:** 2025-06-01

**Authors:** Raanan Meyer, Jayne Caron, Kacey M. Hamilton, Rebecca J. Schneyer, Gabriel Levin, Kelly N. Wright, Matthew T. Siedhoff

**Affiliations:** 1 Division of Minimally Invasive Gynecologic Surgery, Department of Obstetrics and Gynecology, Cedars-Sinai Medical Center, Los Angeles, CA USA; 2 Tel-Aviv, Israel, and The Dr. Pinchas Bornstein Talpiot Medical Leadership Program, Sheba Medical Center, Tel Hashomer, Tel-Aviv University, Ramat-Gan, Israel; 3 Division of Gynecologic Oncology, Jewish General Hospital, McGill University, Quebec, Canada

**Keywords:** Laparoscopy, Obesity, Robotic surgery, Surgical complications, Surgical volume

## Abstract

**Purpose:**

To study the association between body mass index (BMI) and short-term postoperative complications of patients undergoing laparoscopic hysterectomy (LH).

**Study design:**

This is a cohort study of patients who underwent LH for benign conditions. We used prospectively collected data from the American College of Surgeons National Surgical Quality Improvement Program (NSQIP) database from 2012 to 2020. We categorized patients into BMI subgroups and compared 30-day postoperative complication rates, defined by the Clavien–Dindo classification.

**Results:**

206,944 patients met inclusion criteria. In multivariable regression analysis, when comparing lower and higher BMI subgroups, there was a statistically significant increase in any complications [< / ≥ 35.0 kg/m^2^, aOR 95% CI 1.06(1.01–1.10)] and minor complications [< / ≥ 35.2 kg/m^2^, aOR 95% CI 1.13(1.07–1.19)] in the higher BMI group but no differences in major complications. When comparing obesity categories to the normal BMI group, class I, II, and III categories had a lower likelihood of major complications [aOR 95% CI 0.87(0.80–0.93), 0.84(0.77–0.91), 0.82(0.75–0.90), and 0.83(0.75–0.91), respectively] compared to normal weight individuals. Patients in class II and III categories had a higher likelihood of minor complications [aOR 95% CI 1.12(1.03–1.21), and 1.17(1.08–1.28), respectively] compared to normal weight individuals. The mean operative time was significantly longer for each BMI group compared to lower BMI groups (range 115.2–144.5 min, p < 0.05).

**Conclusions:**

Higher BMI was associated with a higher risk of any and minor complications than lower BMI in patients undergoing LH, as well as increased operative time. When comparing specific BMI categories, overweight and obesity categories were associated with lower risks of major complications compared to the normal BMI category.

**What does this study adds to the clinical work?:**

Among women undergoing minimally invasive hysterectomy for benign indications, higher BMI classes were associated with lower risk of short-term postoperative complication compared to the normal BMI class. This information can be used in preoperative planning, counseling, and shared-decision making.

## Introduction

Nearly, one in two adults will be obese by 2030 and nearly one in four adults by 2030 will have severe obesity [1]. As a result, gynecologic surgeons are increasingly likely to encounter patients with higher body mass index (BMI) requiring hysterectomy, raising questions around how to best plan for these operations, counsel patients, choose an appropriate route, and anticipate complications.

ACOG Committee Opinion #701 details that vaginal hysterectomy (VH) should be “the approach of choice whenever feasible” but also describes laparoscopic hysterectomy (LH) as a “preferable alternative to open abdominal hysterectomy (AH) for those patients in whom a vaginal hysterectomy is not indicated or feasible [2].” This same conclusion was found in a recent Cochrane Review which found lower risk of complications with VH and LH compared to AH [3]. A vaginal or laparoscopic approach to hysterectomy rather than abdominal approach results in lower complications, particularly in the subset of patients with class II obesity or greater [4–6]. As physicians become less familiar with the vaginal approach to hysterectomy [7], and as recent research has shown a decreased rate of blood transfusion and decreased length of hospital stay with LH in comparison to VH in patients with larger BMI [8], a laparoscopic approach to hysterectomy in those with larger BMIs is increasingly being utilized as a viable alternative to the vaginal approach, and indeed, the rates of LH continue to increase as the rates of AH and VH decline [9].

There is conflicting literature regarding the relationship between BMI and post-surgical complications in patients undergoing LH which is further complicated by heterogeneity in the definitions of post-surgical complications [10–15]. In particular, two large studies, which used the American College of Surgeons National Surgical Quality Improvement Program (NSQIP) database to assess the association between BMI and postoperative complications following LH, were limited by a lack of consensus on the classifications of postoperative complications [6, 16].

The objective of the study was to better characterize the association between BMI and postoperative complications in a large prospective database.

## Materials and methods

This was a cohort study of prospectively collected data from the NSQIP database of LH cases performed between the years 2012 and 2020. The NSQIP database is a comprehensive, validated database that includes data from approximately 700 participating hospitals [17]. It consists of over 150 variables, collected prospectively, and includes preoperative characteristics, intraoperative factors, and 30-day postoperative outcomes inpatient and outpatient major surgical procedures.

We included hysterectomies performed laparoscopically, including total laparoscopic/robotic hysterectomies (CPT codes 58,570, 58,571, 58,572, 58,573), laparoscopic/robotic supracervical hysterectomies (CPT codes 58,541, 58,542, 58,543, 58,544), and laparoscopic/robotic assisted vaginal hysterectomies (CPT codes 58,550, 58,552, 58,553, 58,554). As robotic surgery is not reported separately from laparoscopic surgery in the NSQIP database, both approaches were included as LHs. VHs and AHs were excluded unless AH was classified as a secondary procedure, in which case it was defined as a conversion to laparotomy. In addition, we excluded cases with missing BMI data, non-elective surgeries, concurrent malignancies, or hysterectomies performed for malignancy, and cases with preoperative sepsis or congestive heart failure.

Patients were categorized into clinically relevant BMI subgroups according to the World Health Organization (WHO): underweight (BMI < 18.5 kg/m^2^), normal BMI (BMI 18.5–24.9 kg/m^2^), overweight (BMI 25–29.9 kg/m^2^), obesity class 1 (BMI 30–34.9 kg/m^2^), obesity class 2 (BMI 35–39.9 kg/m^2^), and obesity class 3 (BMI ≥ 40 kg/m^2^) [18].

We collected baseline and preoperative characteristics, including age, race, tobacco use, and comorbidities such as diabetes mellitus, chronic hypertension, chronic obstructive pulmonary disease, requirement for immunosuppressive therapy, and bleeding disorders. Preoperative parameters included preoperative hematocrit level, the necessity of preoperative transfusion (within 72 h pre-surgery), and American Society of Anesthesiology physical status classification III/IV. Intraoperative variables included surgical indication, hysterectomy approach, uterine weight (< / > 250 g), concomitant procedures performed during the hysterectomy, conversion to laparotomy, wound classification, operative time and hospital length of stay. Postoperative outcomes were classified as major or minor based on the Clavien–Dindo classification system [19]. This classification system has been extensively validated and reports negative events after surgery, ranging from Grade I (any deviation from the normal postoperative course without the need for pharmacological treatment or procedural interventions) to Grade V (death). Grades I–II were considered minor complications, and grades III–V were considered major complications. Major complications included any of the following, occurring within 30 days of surgery: organ space surgical site infection, deep incisional surgical site infection, wound dehiscence, cerebrovascular accident, pulmonary embolism, deep venous thromboembolism, cardiac arrest, myocardial infarction, progressive renal insufficiency, reoperation, reintubation, ventilation > 48 h, sepsis, septic shock, or death. Minor postoperative complications included blood transfusion (within 72 h of surgery start time), superficial surgical site infection, urinary tract infection, acute renal insufficiency, and pneumonia.

The primary outcome was the occurrence of any complication (including minor and major complications), minor and major postoperative complications by BMI category, and the secondary outcome was total operative time.

### Statistics analysis

Univariate analysis was used to compare the six BMI categories. We used Chi-square test and Fisher’s exact test for categorical variables, as appropriate. For continuous variables, we used the Student t-test for comparisons between two groups and the one-way analysis of variance (ANOVA) test for multiple comparisons. To identify between-groups differences in operative time, we used the post hoc Tukey’s honest significant difference test. Categorical variables were reported as proportions and continuous variables as mean (standard deviation). We performed multivariable logistic regression analyses to identify variables independently associated with any, minor, and major postoperative complications. The second multivariable regression analysis used the Kolmogorov–Smirnov test to identify BMI cutoffs for any, minor, and major complications. The following BMI cutoffs were identified: 35.0 (any complications), 39.44 (major complications), 35.16 (minor complication). The second multivariable regression analysis was based on the six BMI groups, where the normal BMI group was used as reference.

The following variables that were identified as statistically significantly different (2-sided *p* value < 0.05) and clinically relevant in univariate analysis were included: age, race, tobacco use, diabetes, chronic hypertension, immunosuppressive therapy, chronic obstructive pulmonary disease, bleeding disorders, ASA classification III/IV, preoperative blood transfusion, surgical indication, hysterectomy type, uterine weight, preoperative hematocrit level, and concomitant surgical procedures. We reported results as adjusted odds ratio (aOR) and 95% confidence interval (CI). Statistical analyses were performed using Software Package for Statistics and Simulation (IBM SPSS version 27, IBM Corp, Armonk, NY) and R Core Team (2022), R Foundation for Statistical Computing, Vienna, Austria.

### Ethical approval

The Institutional Review Board of Cedars-Sinai Medical Center determined that approval was not required for this study (#00003603). The data that support the findings of this study are available on request from the corresponding author.

## Results

**Fig. 1:**
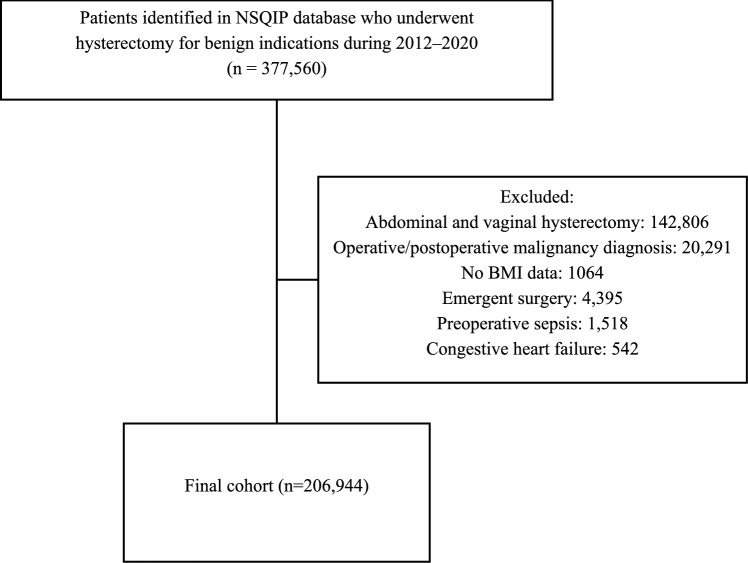
Study population. NSQIP, National Surgical Quality Improvement Program

206,944 patients met the inclusion criteria and comprise the study cohort (Fig. 1), of which 1315 (0.6%) were underweight, 46,209 (22.3%) normal weight, 58,945 (28.5%) overweight, 46,687 (22.6%) obesity class I, 28,307 (13.7%) obesity class II, and 25,481 (12.3%) obesity class III.

Mean age was 46.3 years (Table 1). Those with larger BMIs were more likely to identify as Black (21.0% class III obesity versus 6.2% of normal weight individuals) and less likely to identify as Asian (0.6% class III obesity versus 6.3% of normal weight individuals). Participants in the underweight category were more likely to be smokers (30.7% underweight, 17.0% normal weight) and have a lower preoperative hematocrit (28.2 underweight, 30.97 class III obesity) than those in higher BMI categories. Diabetes, chronic hypertension, and ASA classification of class III/IV were more common in the higher BMI categories compared to those who were underweight or normal weight.

**Table 1:** Baseline characteristics of patients according to body mass index categories

	Underweight < 18.5 (*n* = 1315)	Normal BMI 18.5–24.9 (*n* = 46,209)	Overweight 25–29.9 (*n* = 58,945)	Obesity Class 1 30–34.9 (*n* = 46,687)	Obesity Class 2 35–39.9 (*n* = 28,307)	Obesity Class 3 ≥ 40.0 (*n* = 25,481)	*p* value
Age, years	44.93 (12.57)	46.62 (10.82)	46.93 (10.32)	46.23 (9.79)	45.53 (9.33)	45.29 (9.59)	< 0.001
Race							< 0.001
White	963 (73.2)	33,057 (71.5)	40,394 (68.5)	31,183 (66.8)	18,719 (66.1)	17,109 (67.1)	
Black	82 (6.2)	2802 (6.1)	6766 (11.5)	7519 (16.1)	5453 (19.3)	5358 (21.0)	
Asian	92 (7.0)	2891 (6.3)	2129 (3.6)	889 (1.9)	322 (1.1)	158 (0.6)	
American Indian or Alaska Native	4 (0.3)	181 (0.4)	326 (0.6)	317 (0.7)	215 (0.8)	197 (0.8)	
Native Hawaiian or Other Pacific Islander	3 (0.2)	156 (0.3)	265 (0.4)	250 (0.5)	159 (0.6)	126 (0.5)	
Unknown	171 (13.0)	7122 (15.4)	9065 (15.4)	6529 (14.0)	3439 (12.1)	2533 (9.9)	
Hispanic	68 (5.9)	3336 (8.4)	6891 (13.5)	5763 (14.0)	2930 (11.6)	2262 (9.8)	< 0.001
Smoking	404 (30.7)	7851 (17.0)	9402 (16.0)	7597 (16.3)	4646 (16.4)	3944 (15.5)	< 0.001
Diabetes	21 (1.6)	976 (2.1)	2725 (4.6)	3664 (7.8)	3202 (11.3)	4269 (16.8)	< 0.001
Chronic hypertension	139 (10.6)	5188 (11.2)	11,273 (19.1)	12,442 (26.6)	9319 (32.9)	10,658 (41.8)	< 0.001
Chronic obstructive pulmonary disease	30 (2.3)	291 (0.6)	357 (0.6)	315 (0.7)	255 (0.9)	321 (1.3)	0.001
Immunosuppressive therapy	23 (1.7)	716 (1.5)	857 (1.5)	716 (1.5)	499 (1.8)	464 (1.8)	< 0.001
Bleeding disorders	10 (0.8)	284 (0.6)	385 (0.7)	336 (0.7)	245 (0.9)	297 (1.2)	< 0.001
Preoperative hematocrit level, %	28.20 (37.72)	28.97 (36.35)	29.59 (35.23)	30.26 (34.09)	30.27 (33.85)	30.97 (32.36)	< 0.001
ASA classification III/IV	208 (15.8)	4139 (9.0)	6529 (11.1)	7785 (16.7)	8337 (29.5)	16,141 (63.4)	< 0.001
Preoperative blood transfusion	2 (0.2)	97 (0.2)	133 (0.2)	110 (0.2)	79 (0.3)	79 (0.3)	0.086

BMI, body mass index

Data are n (%) or mean (Standard deviation)

Indications for hysterectomy differed across BMI categories with endometriosis/adenomyosis/pelvic pain being more common in the underweight (22.1%) and normal BMI (17.2%) compared to the obese populations (14.6% class III) (Table 2). Abnormal uterine bleeding was a more common indication for the procedure in the class III obesity cohort (35.1%) than those in the normal weight (23.0%) and underweight categories (20.0%). Pelvic organ prolapse or incontinence was a more common surgical indication in the normal weight group (10.0%) than those with class III obesity (2.1%).

**Table 2:** Surgical characteristics of patients according to body mass index categories

	Underweight < 18.5 (*n* = 1315)	Normal BMI 18.5–24.9 (*n* = 46,209)	Overweight 25–29.9 (*n* = 58,945)	Obesity Class 1 30–34.9 (*n* = 46,687)	Obesity Class 2 35–39.9 (*n* = 28,307)	Obesity Class 3 ≥ 40.0 (*n* = 25,481)	p value
Surgical indication^a^							< 0.001
Uterine fibroids	309 (23.5)	13,818 (29.9)	18,405 (31.2)	14,509 (31.1)	8492 (30.0)	6964 (27.3)	
Endometriosis/adenomyosis/pelvic pain	291 (22.1)	7926 (17.2)	9217 (15.6)	7329 (15.7)	4550 (16.1)	3726 (14.6)	
Pelvic organ prolapse or incontinence	82 (6.2)	4643 (10.0)	5878 (10.0)	3381 (7.2)	1290 (4.6)	526 (2.1)	
Abnormal uterine bleeding	263 (20.0)	10,621 (23.0)	15,194 (25.8)	13,357 (28.6)	8982 (31.7)	8940 (35.1)	
Other indications	370 (28.1)	9201 (19.9)	10,251 (17.4)	8111 (17.4)	4993 (17.6)	5325 (20.9)	
Surgical approach							< 0.001
Total hysterectomy	1208 (91.9)	41,209 (89.2)	52,995 (89.9)	42,645 (91.3)	26,307 (92.9)	23,906 (93.8)	
Supracervical hysterectomy	107 (8.1)	5000 (10.8)	5950 (10.1)	4042 (8.7)	2000 (7.1)	1575 (6.2)	
Uterine weight > 250 g	136 (10.3)	7788 (16.9)	10,789 (18.3)	8722 (18.7)	5337 (18.9)	4997 (19.6)	< 0.001
Concomitant procedures performed during hysterectomy							
Fulguration/excision of ovarian lesions, pelvic viscera, or peritoneal surface	58 (4.4)	1895 (4.1)	2101 (3.6)	1537 (3.3)	930 (3.3)	779 (3.1)	< 0.001
Lysis of adhesions	48 (3.7)	1722 (3.7)	2498 (4.2)	2216 (4.7)	1565 (5.5)	1487 (5.8)	< 0.001
Bladder surgery	56 (4.3)	3087 (6.7)	4353 (7.4)	3117 (6.7)	1642 (5.8)	1147 (4.5)	< 0.001
Ureter procedure	11 (0.8)	356 (0.8)	418 (0.7)	288 (0.6)	180 (0.6)	187 (0.7)	0.063
Ureterolysis	7 (0.5)	207 (0.4)	266 (0.5)	210 (0.4)	136 (0.5)	96 (0.4)	0.580
Appendectomy	14 (1.1)	314 (0.7)	328 (0.6)	191 (0.4)	116 (0.4)	88 (0.3)	< 0.001
Intestinal surgery with enterotomy	8 (0.6)	266 (0.6)	341 (0.6)	323 (0.7)	190 (0.7)	215 (0.8)	< 0.001
Intestinal surgery without enterotomy	33 (2.5)	1140 (2.5)	1653 (2.8)	1329 (2.8)	934 (3.3)	927 (3.6)	< 0.001
Ovarian cystectomy/drainage	1 (0.1)	78 (0.2)	95 (0.2)	80 (0.2)	51 (0.2)	35 (0.1)	0.788
Colpopexy or vaginopexy	78 (5.9)	4193 (9.1)	5189 (8.8)	3133 (6.7)	1311 (4.6)	687 (2.7)	< 0.001
Colporrhaphy	43 (3.3)	2576 (5.6)	3403 (5.8)	2092 (4.5)	955 (3.4)	541 (2.1)	< 0.001
Conversion to laparotomy^b^	1 (0.1)	66 (0.1)	90 (0.2)	89 (0.2)	70 (0.2)	59 (0.2)	0.003
Contaminated or dirty/infected wound class	18 (1.4)	304 (0.7)	400 (0.7)	313 (0.7)	179 (0.6)	187 (0.7)	0.039
Total operative time, minutes	115.15 (53.08)	127.92 (63.33)	132.27 (64.55)	135.20 (65.09)	137.96 (65.51)	144.50 (67.04)	< 0.001
Hospital length of stay, days	0.94 (3.62)	0.84 (2.60)	0.87 (2.53)	0.87 (3.56)	0.89 (2.28)	0.94 (3.48)	0.001

BMI, body mass index

Data are n (%) or mean (standard deviation)

^a^Based on postoperative diagnosis

^b^Based on concurrent exploratory laparotomy or abdominal hysterectomy CPT code

Fulguration/excision of ovarian lesions/pelvic viscera or peritoneal surfaces and appendectomy were more common in the normal weight category (fulguration 4.1%, appendectomy 0.7% normal weight; fulguration 3.1%, appendectomy 0.3% class III obesity). Lysis of adhesions was more common in class III obesity (3.7% normal BMI, 5.8% class III obesity). There were higher rates of colpopexy/vaginopexy and colporrhaphy in the normal BMI category compared to the class III obesity category (9.1% colpopexy/vaginopexy, 5.6% colporrhaphy normal BMI; 2.7% colpopexy/vaginopexy, 2.1% colporrhaphy class III).

Rate of conversion to laparotomy was low (mean 0.12%) across groups, but statistically higher in the BMI class II group compared to normal BMI and overweight (*p* < 0.05). The mean operative time was significantly longer in higher BMI cohorts with the longest average operating time being 144.5 min in the class III obesity cohort and the shortest mean operative time in the underweight category at 115.2 min. In post hoc analysis, a statistically significant difference was found between each BMI group and all other BMI groups (*p* < 0.001).

Table 3 displays the rate of postoperative complications in different BMI categories. Those with class III obesity were more likely to have any surgical complication (8.1% class III, 6.5% normal BMI) and minor surgical complications (5.8% class III, 4.2% normal BMI) compared to the normal BMI group (*p* < 0.005). Major complications were similar between patients with BMI class III (2.9%) and patients with normal BMI (2.8%). Superficial surgical site incision rate increased from the normal BMI group to BMI class III group, with class II and III having statistically more occurrences compared to the normal, overweight, and BMI class I groups (*p* < 0.05). The rate of reoperation was highest in the normal BMI category (1.4% normal weight, 1.0% class III obesity). The rate of sepsis was highest in the class III obesity category (0.5% class III, 0.3% normal weight).

**Table 3:** Postoperative complications of patients according to body mass index categories

	Underweight < 18.5 (n = 1315)	Normal BMI 18.5–24.9 (*n* = 46,209)	Overweight 25–29.9 (*n* = 58,945)	Obesity Class 1 30–34.9 (*n* = 46,687)	Obesity Class 2 35–39.9 (*n* = 28,307)	Obesity Class 3 ≥ 40.0 (*n* = 25,481)	*p* value
Any complication	87 (6.6)	2986 (6.5)	3705 (6.3)	3009 (6.4)	2042 (7.2)	2058 (8.1)	< 0.001
Any major complication	37 (2.8)	1302 (2.8)	1467 (2.5)	1184 (2.5)	743 (2.6)	747 (2.9)	0.001
Any minor complication	62 (4.7)	1926 (4.2)	2503 (4.2)	2056 (4.4)	1411 (5.0)	1472 (5.8)	< 0.001
Minor complication							
Transfusion	22 (1.7)	519 (1.1)	622 (1.1)	454 (1.0)	334 (1.2)	313 (1.2)	0.003
Superficial surgical site infection	15 (1.1)	373 (0.8)	542 (0.9)	498 (1.1)	397 (1.4)	493 (1.9)	< 0.001
Urinary tract infection	24 (1.8)	1041 (2.3)	1348 (2.3)	1116 (2.4)	692 (2.4)	676 (2.7)	0.007
Renal insufficiency	1 (0.1)	2 (0.0)	4 (0.0)	5 (0.0)	0 (0.0)	8 (0.0)	< 0.001
Pneumonia	0 (0.0)	31 (0.1)	54 (0.1)	49 (0.1)	38 (0.1)	42 (0.2)	0.001
Major complication							
Organ space surgical site infection	19 (1.4)	497 (1.1)	609 (1.0)	509 (1.1)	342 (1.2)	335 (1.3)	0.004
Deep incisional surgical site infection	1 (0.1)	65 (0.1)	75 (0.1)	54 (0.1)	49 (0.2)	44 (0.2)	0.203
Wound dehiscence	2 (0.2)	105 (0.2)	110 (0.2)	103 (0.2)	51 (0.2)	74 (0.3)	0.048
Pulmonary embolism	0 (0.0)	75 (0.2)	105 (0.2)	93 (0.2)	55 (0.2)	56 (0.2)	0.290
Cardiac arrest	0 (0.0)	6 (0.0)	5 (0.0)	9 (0.0)	10 (0.0)	4 (0.0)	0.101
Myocardial infarction	0 (0.0)	5 (0.0)	16 (0.0)	8 (0.0)	8 (0.0)	7 (0.0)	0.404
Cerebrovascular accident	0 (0.0)	8 (0.0)	5 (0.0)	5 (0.0)	2 (0.0)	6 (0.0)	0.412
Deep venous thromboembolism	2 (0.2)	52 (0.1)	54 (0.1)	56 (0.1)	38 (0.1)	35 (0.1)	0.401
Ventilation > 48 h	0 (0.0)	0 (0.0)	0 (0.0)	0 (0.0)	0 (0.0)	0 (0.0)	NA
Sepsis	4 (0.3)	133 (0.3)	168 (0.3)	160 (0.3)	101 (0.4)	117 (0.5)	0.001
Septic shock	1 (0.1)	12 (0.0)	12 (0.0)	13 (0.0)	11 (0.0)	15 (0.1)	0.062
Reoperation	17 (1.3)	661 (1.4)	668 (1.1)	505 (1.1)	294 (1.0)	263 (1.0)	< 0.001
Reintubation	0 (0.0)	16 (0.0)	17 (0.0)	22 (0.0)	20 (0.1)	15 (0.1)	0.059
Progressive renal insufficiency	1 (0.1)	14 (0.0)	20 (0.0)	13 (0.0)	8 (0.0)	12 (0.0)	0.704
Death	0 (0.0)	5 (0.0)	6 (0.0)	6 (0.0)	5 (0.0)	6 (0.0)	0.672

BMI, body mass index

Data are n (%)

Table 4 demonstrates multivariable regression analyses of factors associated with postoperative complications. When comparing those with lower BMI and higher BMI, there was a statistically significant increase in any complications [< / ≥ BMI 35.0, aOR 95% CI 1.06 (1.01–1.10)] and minor complications [< / ≥ BMI 35.2, aOR 95% CI 1.13 (1.07–1.19)] in the higher BMI group.

**Table 4:** Multivariable regression analysis of factors associated with postoperative complications

	Any complications^a^ (aOR, 95% CI)	*p* value	Major complications^b^ (aOR, 95% CI)	*p* value	Minor complications^c^ (aOR, 95% CI)	*p* value
Lower BMI^b^	Reference		Reference		Reference	
Higher BMI	1.06 (1.01–1.10)	0.017	0.96 (0.88–1.04)	0.320	1.13 (1.07–1.19)	< 0.001
BMI groups						
Normal BMI (18.5–24.9)	Reference		Reference		Reference	
Underweight (< 18.5)	1.04 (0.82–1.32)	0.748	0.93 (0.67–1.30)	0.670	1.18 (0.89–1.56)	0.242
Overweight (25–29.9)	0.97 (0.92–1.02)	0.219	0.87 (0.80–0.93)	< 0.001	1.02 (0.96–1.09)	0.547
Obesity Class 1 (30–34.9)	0.94 (0.89–1.00)	0.050	0.84 (0.77–0.91)	< 0.001	1.01 (0.94–1.09)	0.761
Obesity Class 2 (35–39.9)	1.01 (0.95–1.08)	0.764	0.82 (0.75–0.90)	< 0.001	1.12 (1.03–1.21)	0.006
Obesity Class 3 (≥ 40.0)	1.04 (0.97–1.12)	0.316	0.83 (0.75–0.91)	< 0.001	1.17 (1.08–1.28)	< 0.001

BMI, body mass index

^a^Adjusted for: age, race, ethnicity, tobacco use, diabetes, chronic hypertension, immunosuppressive therapy, chronic obstructive pulmonary disease, ASA classification III/IV, preoperative blood transfusion, surgical indication, hysterectomy type, uterine weight, concomitant surgical procedures

^b^Adjusted for: age, race, tobacco use, diabetes, chronic hypertension, immunosuppressive therapy, chronic obstructive pulmonary disease, ASA classification III/IV, preoperative blood transfusion, surgical indication, hysterectomy type, uterine weight, concomitant surgical procedures

^c^Kolmogorov–Smirnov test—any complications—35.0, major complications—39.44, minor complication—35.16

When comparing specific classes of obesity to the normal BMI group, there was no longer an increase in any complications seen with higher BMI. Compared to normal BMI, all other BMI groups except the underweight group had lower risks of major complications [overweight- aOR 95% CI 0.87 (0.80–0.93), class I 0.84 (0.77–0.91), class II 0.82 (0.75–0.90), class III 0.83 (0.75–0.91)]. Compared to normal BMI, BMI class II and III had higher risks of minor complications [class II- aOR 95% CI 1.12 (1.03–1.21), class III 1.17 (1.08–1.28)].

## Discussion

When controlling for various comorbid medical conditions and health disparities, higher BMI was associated with a higher risk of all complications and minor complications compared to those with lower BMI in patients undergoing LH for benign conditions. When analyzing specific BMI categories, compared to normal BMI patients, those with higher BMIs had lower risk of major complications, and patients with BMI class II and III had increased risk of minor complications compared to patients with normal BMI. Total operative time increased consistently with BMI categories.

When stratified by BMI category, the results are comparable to a multicenter study that found no association between BMI category and the risk of any complications [10]. In contrast, a large institutional study showed increasing post-op complications with LH in those with higher BMI, with the highest complication rate seen in patients with BMI > 40 [12]. However, that study utilized a composite index score that included operating room time and length of stay in addition to severe post-op complications. In a 2015 study utilizing the NSQIP database, there was no increase in post-surgical complications associated with LH. However, the study was limited by a lack of controls for concomitant procedures and did not stratify complications according to the Clavien–Dindo scale [6].

When stratifying BMI categories into two groups, no difference was found in major complications. A systematic review identified higher BMI as a risk factor for postoperative surgical complications in patients undergoing LH. The most common cutoff value used in the included studies was BMI ≥ 30 kg/m^2^, stratification by BMI was not reported, and complications severity definitions differed between included studies [13, 15]. In contrast and in line with this study’s results, a large retrospective cohort study utilizing English National Health Services data did not find an association between higher BMI and major complications with LH [14].

Interestingly, a reverse association between higher BMI categories and the risk of major complications was found. This association was not reported in previous studies. One possible explanation may be the obesity paradox [20]. Reported in various metabolic and cardiovascular diseases, those with mild obesity or overweight status actually have better health outcomes than those with normal weight BMI. A proposed mechanism of action of this is that adiposity results in enhanced glucose availability and activation of the immune response which can enhance recovery after periods of acute critical illness [21]. Another possibility is that, as surgical volume declines for generalist OB/GYNs [22], more difficult cases, such as those with significantly high BMI, are being referred to those with high volume and/or subspecialty training in minimally invasive gynecologic surgery (MIGS), as higher surgical volume and fellowship training are associated with a decreased risk of complications [23–26].

This study, consistent with prior literature, demonstrated longer operating times for each additional BMI category. This finding makes plausible sense as increasing adiposity can make entry, patient positioning, and closure more challenging in laparoscopic cases. However, as prior literature has shown, this increase in operative time should not dissuade surgeons from utilizing a laparoscopic approach as the increase in operative time was seen in VH, LH, and AH in higher BMI patients [6]. In one study that modeled risks of complications between LH and AH, under no scenario did increased operative time outweigh the benefits of a laparoscopic approach [27].

Clinically, this study underscores the importance of considering patient’s BMI when counseling patients regarding their risk of postoperative complications following LH. Clinicians can use the stratification based on BMI category for reassurance regarding major postoperative complications among patients with higher BMI categories, while acknowledging a possible higher risk of minor complications. Patients with elevated BMI in need of LH may benefit from referral to a MIGS or otherwise high-volume surgeons.

Regarding health policy, the consistent finding across the wealth of literature analyzing BMI and LH of longer operative times suggests that considerations of BMI should be considered when allocating physician surgical reimbursement [12].

Future studies in this area are needed to elucidate the underlying factors contributing to the disparity in outcomes among different BMI subgroups. Further research analyzing the obesity paradox in gynecologic populations is warranted as current data are limited [28] Stratification by complication severity, as used in the current study, is paramount.

This study had several strengths in building on previous literature examining the relationship between BMI and surgical outcomes following LHs. By focusing exclusively on LHs utilizing the NSQIP database, a large sample size was included, allowing to detect small, but meaningful, differences between groups. The categorization into total and supracervical hysterectomies, a factor included in the multivariable regression analysis, is another strength of this study. In addition, controlling for concomitant surgical procedures when examining rates of post-op complications across BMI categories adds to the robustness of the findings.

There are several limitations to this study. Factors not included in the NSQIP database, such as patient’s prior surgical history, insurance status, obstetric history, and socioeconomic status, were not controlled for in the analysis. Importantly, the NSQIP database does not provide information on surgeon skills, volume or subspecialty training, which may limit the ability to detect changes in complications across BMI categories. It is assumed that patients with higher BMI may have been protected against complications by referral to a MIGS/high-volume surgeon. In addition, cases without reported BMI were excluded from the analysis, potentially introducing bias. Importantly, the NSQIP database only looks at 30 days post-operatively and thus longer-term outcomes and complications are not captured in this study. In addition, the NSQIP database only allows one ICD code to be applied per case. Thus, the analysis does not allow to control for patients who may have had multiple surgical indications, although controlling for additional concomitant procedures may mitigate this limitation. Furthermore, this study utilizes BMI to assess body fat composition, which is increasingly being viewed as a flawed metric when attempting to assess visceral fat and perioperative risk [29]. Lastly, NSQIP does not allow to distinguish between robot-assisted versus traditional laparoscopy, although current literature does not demonstrate a significant difference in outcomes between these two approaches [30].

In conclusion, while higher BMI was associated with a higher risk of complications compared to lower BMI, these distinctions were reversed when analyzing major complications. Higher BMI experienced longer surgical times.

## Data Availability

The data that support the findings of this study are available from the corresponding author upon reasonable request.

## Abbreviations

BMI: Body mass index
VH: Vaginal hysterectomy
LH: Laparoscopic hysterectomy
AH: Abdominal hysterectomy
NSQIP: National Surgical Quality Improvement Program
CPT: Current Procedural Terminology
